# CATO: The Clone Alignment Tool

**DOI:** 10.1371/journal.pone.0159586

**Published:** 2016-07-26

**Authors:** Peter V. Henstock, Peter LaPan

**Affiliations:** 1 Pfizer R&D Business Technologies, 1 Burtt Road, Andover, MA, United States of America; 2 Pfizer Global Biotherapeutic Technologies, 700 Main Street, Cambridge, MA, United States of America; Huazhong University of Science and Technology, CHINA

## Abstract

High-throughput cloning efforts produce large numbers of sequences that need to be aligned, edited, compared with reference sequences, and organized as files and selected clones. Different pieces of software are typically required to perform each of these tasks. We have designed a single piece of software, CATO, the Clone Alignment Tool, that allows a user to align, evaluate, edit, and select clone sequences based on comparisons to reference sequences. The input and output are designed to be compatible with standard data formats, and thus suitable for integration into a clone processing pipeline. CATO provides both sequence alignment and visualizations to facilitate the analysis of cloning experiments. The alignment algorithm matches each of the relevant candidate sequences against each reference sequence. The visualization portion displays three levels of matching: 1) a top-level summary of the top candidate sequences aligned to each reference sequence, 2) a focused alignment view with the nucleotides of matched sequences displayed against one reference sequence, and 3) a pair-wise alignment of a single reference and candidate sequence pair. Users can select the minimum matching criteria for valid clones, edit or swap reference sequences, and export the results to a summary file as part of the high-throughput cloning workflow.

## Introduction

Subcloning of restriction fragments and PCR products is a common technique employed in many academic and industrial laboratories[1]. The application of automation and high-throughput techniques to these processes results in a large number of reference sequences that need to be compared to even larger numbers of candidate clone sequences. To identify the candidate sequences corresponding to each reference sequence, multiple sequence alignments are often performed for each reference sequence. Although a number of software applications exist for visualizing aligned sequences, they are not focused on high-throughput cloning and are thus difficult to use efficiently with large batches of sequences. A full analysis of clone sequence data requires multiple modes, including sequence alignment, editing, visualization, file manipulations and clone selection. Several different pieces of software are typically required to perform each of these, but the software tool described here, CATO, efficiently performs all these functions. The recently released ANTICALIgn software [2] similarly aims to bring together multiple tools for protein engineering, but differs from CATO in that it focuses on only a single reference sequence and corresponding aligned clones at one time. When supplied with reference and candidate sequences, CATO can perform a bulk comparison, aligning every candidate sequence with every reference sequence. Visualizations and metrics are provided to assist in identifying the best match candidates, after which the CATO session can be saved and shared between scientists. The final output is a compilation of candidates meeting user-specified threshold metrics that can be exported for downstream analysis.

Our main use case comes from the high-throughput antibody discovery process. Researchers often begin with a collection of truncated antibody fragments (known as single-chain variable fragments, or scFv) in bacterial expression vectors, which are isolated based on functional testing and then sequenced[3, 4], providing the reference sequences in this case. In order to test these molecules in their final therapeutic format, full-length immunoglobulin G (IgG), researchers must generate mammalian expression vectors in which two variable fragment cassettes are subcloned in-frame with genes encoding the remainder of the full IgG heavy and light chains. In this case, CATO serves two purposes: to allow sequence verification of large numbers of antibody-encoding plasmids by rapidly matching IgG heavy and light chain subclones with the sequences of the scFv from which they came, and to resolve ambiguities that may exist in the original scFv sequences by allowing rapid comparison with subclones, which often have higher-quality sequence.

A second use case is in introduction of specific point mutations during protein engineering. Amino acid sequence homology or protein secondary structure are used to identify specific residues to be mutated, either individually or in combination, using oligonucleotide-directed mutagenesis[5, 6]. Identification of variants that have incorporated the designed mutations requires comparison of the sequences of isolated clones against the intended sequences of the variants, which can be a tedious and error-prone task when conducted with standard sequence alignment software. CATO allows rapid identification of correct variants from large-scale mutagenesis experiments, whether individual mutagenesis reactions are kept separate or are pooled.

CATO is written in Java and will run on all major operating systems. The CATO distribution includes JAligner which provides the alignment algorithm, and JGoodies which provides the look-and-feel. Using a 64-bit Windows 8 laptop with a 1.3 GHz Intel i5-4300 CPU and 8GB RAM, CATO can analyze 200 reference sequences and 800 candidate sequences in under 10 minutes.

## Results and Discussion

CATO provides a simple user interface for the high-throughput cloning and sequence analysis steps including alignment, scoring, visualization, annotation and editing. Standard multiple sequence alignment and scoring approaches could be set up for each reference sequence; standard alignment viewers and editors could provide functionality. However, the ability to easily manage hundreds of separate alignments, the specialized visualization to quickly identify exact or poor matches, and the custom tools to correct for errors that commonly occur in cloning are the main benefits of CATO. The following sections provide details on the methods encompassed by CATO.

### Computational Algorithms

The algorithmic goal is to identify contiguous regions of exact matches between each candidate sequence and the corresponding reference sequence. An exact match of 100% identity between cloning sites indicates that a clone sequence has been amplified with high fidelity and that the junctions of fused DNA fragments have the expected sequences. In theory, a simple string match could have been used, but the effectiveness of this approach is limited by the inherent variability caused by unknown sequencing reaction starting points, perfect but curtailed sequence copies, mismatches, and unknown sequence reads designated by ‘N’s. The solution was to use a standard sequence alignment algorithm to identify the closely-aligned region.

Sequence alignment algorithms are generally divided into two general categories: pair-wise matching and multiple sequence alignment. The former category includes the common BLAST [7] and Smith-Waterman [8] algorithms that can be used to align each candidate sequence against the reference sequence. The latter category aligns multiple sequences against each other simultaneously and includes the ClustalW [9], Muscle [10], and T-Coffee [11] approaches. While both categories are applicable in this context, we elected to use the pair-wise Smith-Waterman algorithm with Gotoh’s affine gap penalty [12] available in the JAligner package [13]. The gap penalties are configurable by the user and are set by default to 0.5. Pair-wise alignment algorithms facilitate an alignment of clone sequences against the standard reference sequence and are more computationally efficient than the multiple sequence alignment approaches. Smith-Waterman met these objectives quite well and was readily available in Java for a stand-alone program. Furthermore, in finding local alignments, Smith-Waterman successfully identified stretches of common alignment between the reference and clones, while maintaining the integrity of the reference sequence. Smith-Waterman also facilitated an identification of mismatched pairs often caused by an incorrect reference sequence.

### Visualization

In addition to multiple sequence alignment algorithms, there are multiple approaches to sequence visualization, each with its own visual representation. Pair-wise sequence alignments are commonly represented with a two-row alignment showing matches, mismatches, and gaps. Due to the sequence length, the two rows are often wrapped onto multiple lines. For multiple sequence alignment, there are a number of separate viewers including PFAAT [14] and Jalview [15], with a more complete list referenced in [16]. To summarize the alignments to the reference sequence, several bioinformatic and other numeric scores are frequently included to represent the quality of the match and key values such as the extent of the perfectly-matched regions [17, 18]. CATO uses the percent of the reference sequence found, the length of the longest continuous match, the percent match, and the Smith-Waterman local alignment score with gap penalties for starting and extending gaps in the alignment.

**Fig 1:**
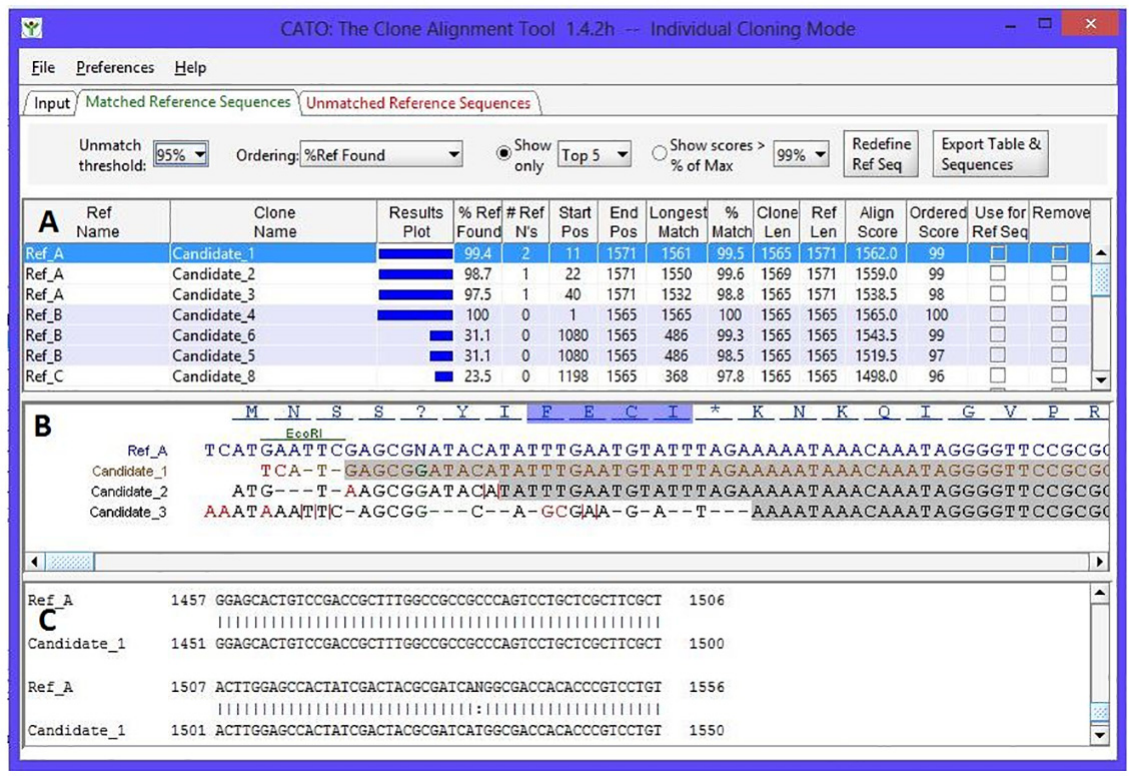
Main CATO interface. Results view with 3 panels showing the summary of reference sequences and matched candidate sequences are displayed in panel A. A focused view of a single reference sequence and associated clone candidate sequences is shown in panel B, with the highlighted candidate from the top panel colored brown. The top blue amino acid sequence has motifs highlighted. The blue nucleotide sequence is the reference sequence with restriction sites indicated. Candidate mismatch nucleotides are red, gaps indicated with a dash, and vertical red bars indicate 1 or more nucleotides missing from the reference. A grey background highlights the longest contiguous match with the reference sequence. A single reference-candidate sequence pair-wise alignment is in panel C.

The CATO visualization includes three different views to rapidly provide the user with insight into the data. The CATO system is essentially a hybrid between a single alignment BLAST type of matching and a multiple sequence alignment approach. That is, CATO does not use a multiple sequence alignment approach but produces such a view by performing multiple pair-wise alignments between the reference sequence and multiple candidate sequences and then displaying them together. The top panel A of Fig 1 shows a list of the reference sequences, each with its closest corresponding matching candidate sequences. Each pair of reference and candidate sequences has several recorded statistics representing a combination of standard metrics as found in [19] and measures specific to the objective of CATO including:

- a graphical display showing the portion of the reference sequence matched
- the percent of the reference sequence perfectly matched by a contiguous clone sequence
- the number of ambiguities (undetermined nucleic acids from the sequencing process)
- the length, start, and stop position of the match candidate sequence relative to the reference sequence
- the percent of the reference sequence matched using the Smith-Waterman local alignment score with the gap penalty (non-contiguous)

Panel B of Fig 1 is a multiple sequence alignment view of the top candidate sequences against the single reference sequence selected in panel A. The first nucleotide sequence row (with associated amino acid sequence when calculated) is always the reference sequence. The candidate sequence selected for detailed comparison to this reference sequence is colored brown in panel B. Since one of the requirements is to align against the reference sequence, gaps in the reference sequence indicative of extra insertions in the candidate sequence are not shown in this view. Instead, the reference sequence is displayed without spaces, but the corresponding candidate sequence has red vertical bars inserted to indicate the mismatch. Gaps in the candidate sequence relative to the reference sequence are represented with the standard dash notation. To allow rapid examination of the full set of insertions and deletions, the gapped alignment with a candidate sequence can be viewed in panel C (see below). Mismatched nucleotides are displayed in panel B in a different color (red by default), and the columns where the reference sequence contains an ambiguity are displayed in a separate color and denoted with the “N” symbol. Many multiple sequence alignments such as those for evolutionary trees display each amino acid or nucleic acid in a different color to quickly identify the matches. CATO is designed to emphasize perfect matches rather than nucleic acid patterns, so the colored (default gray) box in panel B spans the longest contiguous exact match region for each candidate sequence against the reference sequence. The dimensions of matching region depicted with the gray box correspond to the blue match bars shown in panel A.

Panel C of Fig 1 is simply the pair-wise match between the reference and a single selected candidate sequence. The JAligner software takes two sequences, creates a matched region of two sequences, and produces a view that includes matches, mismatches, and gaps. It is often the case that the central region of the sequences match but the 5’ and 3’ regions do not match. In such cases, only the central matching region will be shown in the pair-wise alignment view.

Depending on the number of clones included in a particular experiment, there may be an unwieldy number of clones associated with a reference sequence. With drop-down menus above panel A, the user can specify either a maximum limit of clones to display against each reference sequence, or restrict the clones displayed to those with a certain percentage of match against the reference sequence.

### Candidate selection

For the process of aligning cloned candidate sequences against the reference sequences, there are two goals. First, the user wants to identify a perfect match candidate for each reference sequence. In practice, sequences often have ambiguities, so multiple candidates may be selected for resequencing. The second goal is identifying reference sequences for which there are no matching cloned candidate sequences, which is particularly important in identifying cloning failures when large numbers of genes are being subcloned from a single pool. Since these two cases are quite distinct, the reference sequences and corresponding candidate sequences are separated onto two different panels based on a user-specified threshold fraction of the reference sequence matched called the “Unmatch threshold.” From our experience, cloned sequences tend to map 70–90% of the reference sequence whereas sequences with poor matches comprise 20–50% of the total. However, if the reference sequence contains an excess of vector sequence, the percent match to reference for a perfect insert match would be much lower.

### Bulk versus individual cloning

Cloning experiments are performed in one of two modes: bulk or individual. The bulk process represents the process we have described thus far in the text where all the candidate sequences are matched against each reference sequence. In the individual cloning process as displayed in Fig 1, each candidate clone should only be compared to a specified reference sequence. CATO offers an optional two-column association file on the input form to perform this mapping. Apart from the computational issues, it could be argued that the bulk approach should be sufficient for both processes. However, if the references are similar, with only minor variations in the clone sequences, there may often be incorrect associations. The comparison of clones to individual references is also useful in a sequence confirmation operation, when users are comparing the sequences of replicate samples against a possibly imperfect reference.

### Sequence annotation

**Fig 2:**
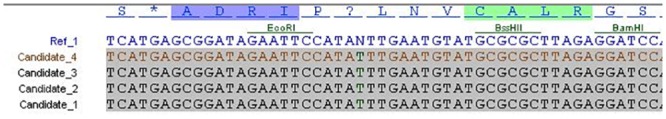
View restriction sites and amino acid motifs. Zoomed view of the middle panel showing the EcoRI, BssHII, and BamHI restriction enzyme sites and the amino acids with the reference frame motif in purple and a second motif shown in green. The ambiguous nucleotide (“N”) in the reference sequence can be inferred to be a “T” based on the consensus amongst the candidates.

To assist users in evaluating sequence alignments and errors, panel B of Fig 1 can include two additional pieces of information (Fig 2). First, restriction enzymes sites within the reference sequence can be included. Since such enzymes are specific to the type of cloning, the user has the option of providing a file listing the restriction enzyme names and sequences to be displayed in this view. Second, the corresponding amino acids can be displayed also above the reference sequences to show the resulting protein translation. To find the correct reading frame, the user must provide an ordered set of amino acid motifs, with the first match defining the reading frame. The first (purple) and all subsequent matching motifs (green) with the same reading frame are displayed as boxes using two different colors. Codons containing an ambiguous nucleotide are converted into a “?” amino acid for the display.

### Changing the reference sequence

**Fig 3:**
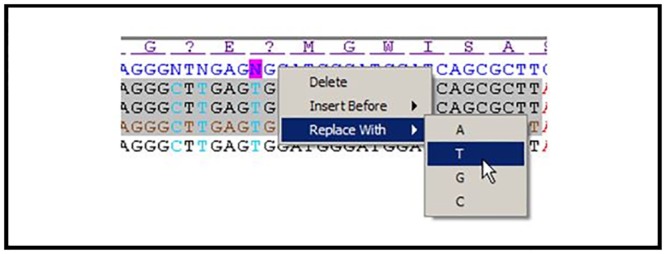
Sequences can be edited. Right clicking on the reference sequence (N) allows the full range of sequence modifications.

In high-throughput operations, reference sequences may be based on a single read in one direction. This first sequence read becomes the reference sequence even if it is of low quality. With repeated resequencing, the CATO user can take advantage of sequence information in the collection of subclones to resolve ambiguities in the original sequence and to create a corrected reference sequence for future use. In examining a reference sequence aligned to multiple clone sequences, errors can be quickly identified (Fig 2). The simplest change is to correct an ambiguity in the reference sequence using multiple cross-validating candidate sequences. It is also possible to make the full set of single-base substitutions in the reference sequence, including insertion and deletion (Fig 3). Although multiple edits can be made, the user will be prompted to rerun the full processing when the editing is complete to ensure reference candidate sequence assignments are accurate. Each rerun of the process generates a new file set, keeping the original files unmodified.

**Fig 4:**
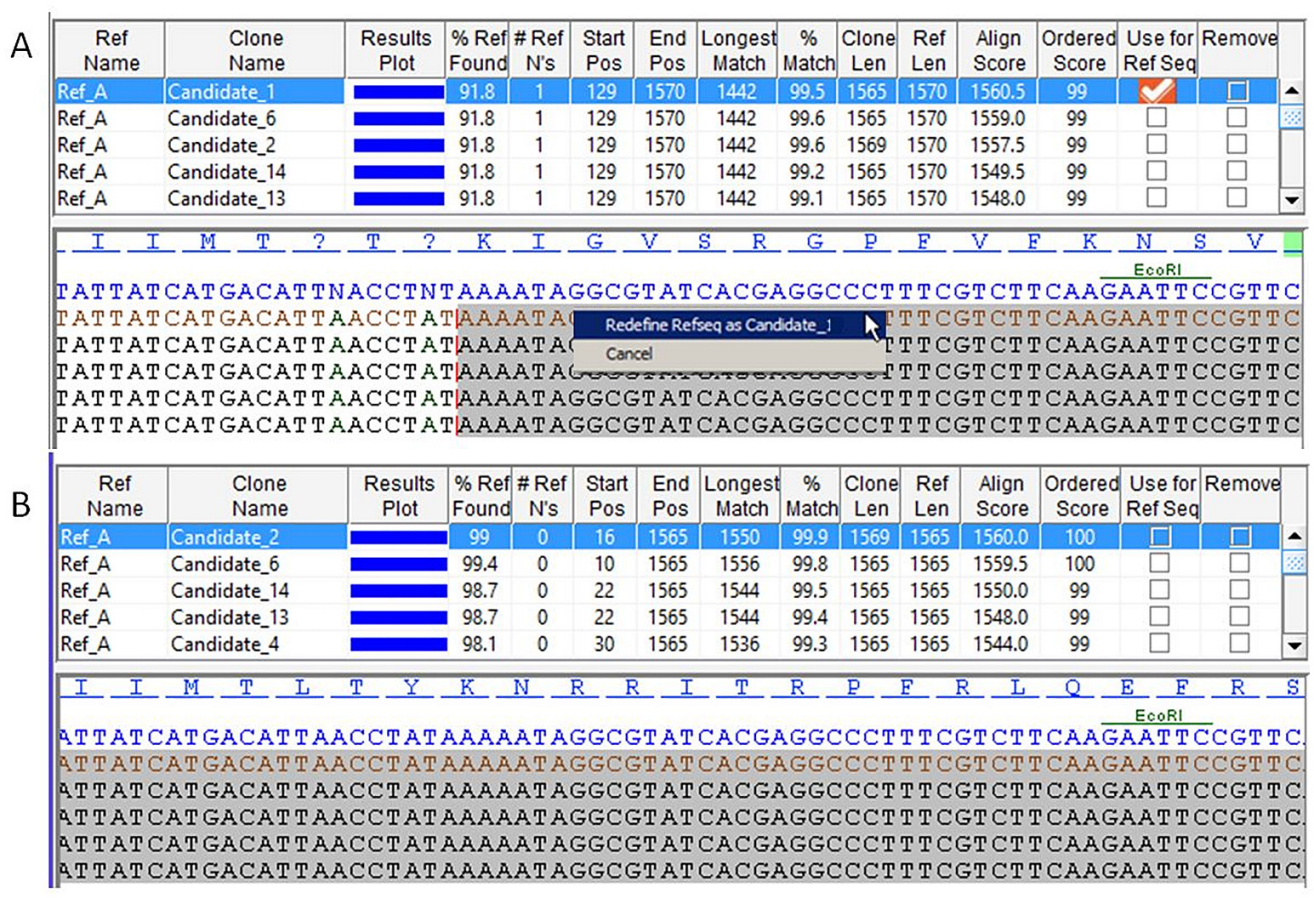
Change the reference sequence. Right-clicking a candidate sequence (A) results in an orange check mark in the “Use for Ref Seq” column. After such changes, the alignment needs to be saved and rerun, after which the selected clone sequence will become a reference sequence and the previous reference sequence will become a candidate sequence. (B) The same view as (A) with the new reference sequence now being utilized. Ambiguities are resolved and the original single-base deletion is restored resulting in correction of the reading frame.

Should the reference sequence contain many ambiguities relative to a candidate subclone, the user may wish to replace the reference sequence with one of the candidate sequences. This can be performed using either the check-boxes in the table view (“Use for Ref Seq”) or right-clicking in the middle panel (“Redefine Refseq as…”) as shown in Fig 4. The result effectively switches the clone and reference sequence. The new reference sequence can then be manually edited if needed, after which the data will have to be reprocessed.

## Conclusion

The CATO system has been used for over a year to facilitate clone discovery within Pfizer’s Global Biotherapeutics antibody drug discovery programs. Utilization of CATO has removed an analysis bottleneck, streamlined and standardized a process that previously could vary significantly between users, and it has eliminated many sources of clerical error that accompany manual sequence manipulation. With its standard FASTA input and output files, it can be readily used as a standalone tool or integrated into any workflow to provide user-guided sequence alignments.
